# μ-Opioid receptor desensitization: homologous or heterologous?

**DOI:** 10.1111/ejn.12003

**Published:** 2012-09-24

**Authors:** Javier Llorente, Janet D Lowe, Helen S Sanderson, Elena Tsisanova, Eamonn Kelly, Graeme Henderson, Chris P Bailey

**Affiliations:** 1 School of Physiology & Pharmacology, University of BristolBristol, UK; 2 Department of Pharmacy & Pharmacology, University of BathBath, UK

**Keywords:** desensitization, GPCR, opioid receptor, opioids, rat

## Abstract

There is considerable controversy over whether μ-opioid receptor (MOPr) desensitization is homologous or heterologous and over the mechanisms underlying such desensitization. In different cell types MOPr desensitization has been reported to involve receptor phosphorylation by various kinases, including G-protein-coupled receptor kinases (GRKs), second messenger and other kinases as well as perturbation of the MOPr effector pathway by GRK sequestration of G protein βγ subunits or ion channel modulation. Here we report that in brainstem locus coeruleus (LC) neurons prepared from relatively mature rats (5–8 weeks old) rapid MOPr desensitization induced by the high-efficacy opioid peptides methionine enkephalin and DAMGO was homologous and not heterologous to α_2_-adrenoceptors and somatostatin SST_2_ receptors. Given that these receptors all couple through G proteins to the same set of G-protein inwardly rectifying (GIRK) channels it is unlikely therefore that in mature neurons MOPr desensitization involves G protein βγ subunit sequestration or ion channel modulation. In contrast, in slices from immature animals (less than postnatal day 20), MOPr desensitization was observed to be heterologous and could be downstream of the receptor. Heterologous MOPr desensitization was not dependent on protein kinase C or c-Jun N-terminal kinase activity, but the change from heterologous to homologous desensitization with age was correlated with a decrease in the expression levels of GRK2 in the LC and other brain regions. The observation that the mechanisms underlying MOPr desensitization change with neuronal development is important when extrapolating to the mature brain results obtained from experiments on expression systems, cell lines and immature neuronal preparations.

## Introduction

There is now substantial evidence that the mechanisms underlying μ-opioid receptor (MOPr) desensitization are agonist-dependent and that high-efficacy opioid peptide agonists induce desensitization in a manner different from low-efficacy alkaloids such as morphine (Johnson *et al*., 2006; Bailey *et al*., 2006, 2009; Kelly *et al*., 2008; Melief *et al*., 2010; Lau *et al*., 2011; Grecksch *et al*., #b^500^). However, the published literature on how high-efficacy opioid peptides such as methionine enkephalin (ME) and d-Ala^2^, *N*-MePhe^4^, Gly-ol-enkephalin (DAMGO) desensitize MOPr is both confusing and contradictory (reviewed by Dang & Christie, 2012). Even in the same neuronal population, rat brainstem locus coeruleus (LC), there is disagreement about whether MOPr desensitization induced by ME and DAMGO is predominately homologous (Christie *et al*., 1987; Harris & Williams, 1991; Osborne & Williams, 1995; Alvarez *et al*., 2002; Bailey *et al*., 2004) or heterologous (Blanchet & Lüscher, 2002) and whether it involves a G-protein coupled receptor kinase (GRK) and arrestins (Bailey *et al*., 2009; Dang *et al*., 2009; Quillinan *et al*., 2011; Arttamangkul *et al*., 2012).

Raveh *et al*. (2010) have presented evidence that in HEK 293 cells ME-induced MOPr desensitization resulted not from the canonical mechanism of G protein-coupled receptor (GPCR) desensitization involving receptor phosphorylation by GRK and arrestin binding, but from sequestration by GRK of the free G protein βγ subunits required to activate the downstream signalling through G-protein activated inwardly rectifying potassium (GIRK) channels. Desensitization mediated downstream of the receptor is likely to result in heterologous desensitization with other GPCRs utilizing the same effector pathway (Blanchet & Lüscher, 2002; Tan *et al*., 2003). An important question about the phenomenon of desensitization occurring downstream of the receptor is whether it occurs with endogenous G_i_/G_o_-coupled receptors and GIRK channels in mature neurons.

In LC neurons activation of MOPrs, α_2_-adrenoceptors and SST_2_ receptors activates GIRK channels (North & Williams, 1985; Connor *et al*., 1997). The GIRK channel current in response to simultaneous MOPr and α_2_-adrenoceptor activation did not exceed the maximum current in response to MOPr activation alone, indicating that these receptors couple through G_i_/G_o_ proteins to the same set of GIRK channels. North & Williams (1985) concluded that in LC neurons the pool of GIRK channels was limiting. More recently, however, Levitt *et al*. (2011), studying MOPr inhibition of adenylyl cyclase activity and GTPγS binding in SH-SY5Y cells, have suggested that in those cells it is the available G protein pool that is limiting.

In the present study we have examined the rapid desensitization of GIRK channel currents in response to activation of MOPrs, α_2_-adrenoceptors and SST_2_ receptors in LC neurons. We found no evidence to support the view that in mature LC neurons rapid MOPr desensitization involves effects at the level of the G-protein or GIRK channel. Furthermore, although MOPr desensitization in LC neurons from mature rats was homologous, in LC neurons from immature rats it was heterologous and may involve changes in the effector pathway.

## Materials and methods

### Brain slice preparation

All experiments were conducted on brain slices from 5- to 8-week-old animals except where stated otherwise. Male Wistar rats were killed either by cervical dislocation or, for experiments comparing responses in slices from young (less than postnatal day P20) and older (5–8 weeks) rats, by decapitation under ketamine- (160 mg/kg) and xylazine- (20 mg/kg) induced anaesthesia, and the brains removed and submerged in ice-cold cutting solution containing (in mm): 20 NaCl, 2.5 KCl, 0.5 CaCl_2_, 7 MgCl_2_, 1.25 NaH_2_PO_4_, 85 sucrose, 25 d-glucose and 60 NaHCO_3_, saturated with 95% O_2_/5% CO_2_. Horizontal slices (250 μm thick) containing the LC were prepared using a vibroslice (Leica) (Bailey *et al*., 2009). Immediately upon cutting, slices were submerged in an artificial cerebrospinal fluid (aCSF) containing (in mm): 126 NaCl, 2.5 KCl, 1.2 MgCl_2_, 2.4 CaCl_2_, 1.2 NaH_2_PO_4_, 11.1 d-glucose, 21.4 NaHCO_3_ and 0.1 ascorbic acid, saturated with 95% O_2_/5% CO_2_ at 34 °C, and were left to equilibrate for at least 1 h. All experiments were performed in accordance with the UK Animals (Scientific Procedures) Act of 1986, the European Communities Council Directive of 1986 (86/609/EEC), and the Universities of Bristol and Bath ethical review documents and had institutional approval from both the Universities of Bristol and Bath.

### Whole-cell patch-clamp recordings

Slices were submerged in a slice chamber (0.5 mL) and superfused (2.5–3 mL/min) with aCSF at 33–34 °C. LC neurons were visualized by Nomarski optics, and individual cell somata were cleaned by gentle flow of aCSF from a pipette. Whole-cell voltage-clamp recordings (*V*_h_ 60 mV) were made using electrodes (3–6 MΩ) filled with (in mm): 115 potassium gluconate, 10 HEPES, 11 EGTA, 2 MgCl_2_, 10 NaCl, 2 MgATP and 0.25 Na_2_GTP, pH 7.3 (osmolarity 270 mOsm). Recordings of whole-cell current were filtered at 2 kHz using an Axopatch 200B amplifier and analysed offline using pClamp. All drugs were applied in the superfusing solution in known concentrations. In those experiments in which α_2_-adrenoceptor responses were studied, noradrenaline (NA) was applied in the presence of prazosin (1 μm) and cocaine (3 μm).

### Determination of GRK2 expression levels by Western blotting

Brain regions (cortex, hippocampus, LC and striatum) were rapidly dissected from the brains of young and mature rats and homogenized using a hand-held dounce in ice-cold phosphate-buffered saline with 1% (w/v) sodium dodecyl sulfate (SDS) and the samples heated at 95 °C for 10 min. Cell debris and nuclei were then removed by centrifugation for 5 min at 17,000 g at 4 °C in a microcentrifuge. Fifteen-microgram samples of the resulting supernatant were subjected to SDS polyacrylamide gel electrophoresis. Proteins were blotted onto polyvinylidene fluoride (PVDF) membranes and probed with rabbit polyclonal antibodies for GRK2 (C-15; dilution 1 : 1000; Santa Cruz Biotechnology, Santa Cruz, CA, USA) and pan-arrestin (1 : 1000; Ab2194, Abcam, Cambridge, UK). Following subsequent incubation with a horseradish peroxidase -linked secondary antibody (NA934V, donkey anti-rabbit; dilution 1 : 7500; GE Heatlthcare, Little Chalfont, UK), bands were visualized by enhanced chemiluminescence (ECL) with SuperSignal West Dura Chemiluminescent Substrate (Thermo Scientific). Membranes were reprobed with a mouse anti-tubulin antibody (1 : 10 000, T6074, Sigma, Gillingham, UK) and visualized following incubation with a horseradish peroxidase-linked secondary antibody with ECL (GE Healthcare). Densitometry of the GRK2 and tubulin bands was undertaken using ImageJ. Duplicate values were taken for each sample and then averaged. GRK2 levels were then normalized against corresponding tubulin values.

### Statistics

Electrophysiological data were analysed by one- or two-tailed Student's *t*-tests as appropriate, using Prism5 (Graphpad Software Inc., La Jolla, CA, USA). Levels of GRK expression between young and older animals, as assessed by densitometry, were compared using a one-sample *t*-test. In each experiment, GRK2 expression in brain regions from the younger animals were taken as 100%. Differences were assumed to be significant at *P* < 0.05.

## Results

**FIG. 1:**
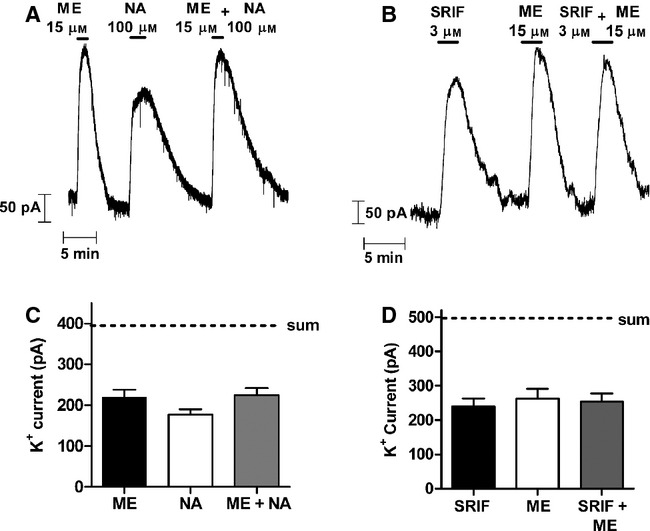
GIRK channel currents in mature rat LC neurons evoked by maximally effective concentrations of ME, NA and SRIF do not add together. (A,B) Outward potassium currents recorded from single LC neurons in each case in response to application of maximally effective concentrations of ME, NA and SRIF. (C, D) Pooled data from experiments as illustrated in A and B, showing that the current evoked by ME (*n* = 6) and NA (*n* = 6) or ME (*n* = 4) and SRIF (*n* = 4) in combination was not greater than that evoked by ME alone. The estimated level of current if the responses had summated is indicated by the dashed line (sum).

North & Williams (1985) first reported that in LC neurons the GIRK current in response to simultaneous MOPr and α_2_-adrenoceptor activation in total did not exceed the maximum current evoked by activation of MOPr alone. We have extended that observation to include SST_2_ receptors. In LC neurons the current in response to a maximally effective concentration of the MOPr endogenous agonist ME (15 μm) was always slightly greater than the maximum current evoked through α_2_-adrenoceptors by NA (100 μm; Fig. 1A and C). When LC neurons were exposed at the same time to maximally effective concentrations of ME and NA the amplitude of the outward GIRK channel current was not greater than that activated by ME alone, i.e. the currents did not summate (*t*_10_ = 0.19, *P* = 0.85). Somatostatin (somatotropin release-inhibiting factor, SRIF) acting on SST_2_ receptors also activates GIRK channel current in LC neurons (Chessell *et al*., 1996; Connor *et al*., 1997). The maximum current evoked by SRIF (3 μm) was similar to the maximum evoked by ME and the currents evoked by both did not summate (Fig. 1B and D) (*t*_6_ = 0.46, *P* = 0.66). These observations suggest that in the LC MOPrs, α_2_-adrenoceptors and SST_2_ receptors couple to the same set of GIRK channels and that either the levels of the G-protein or GIRK channels are the limiting factor in response amplitude.

**FIG. 2:**
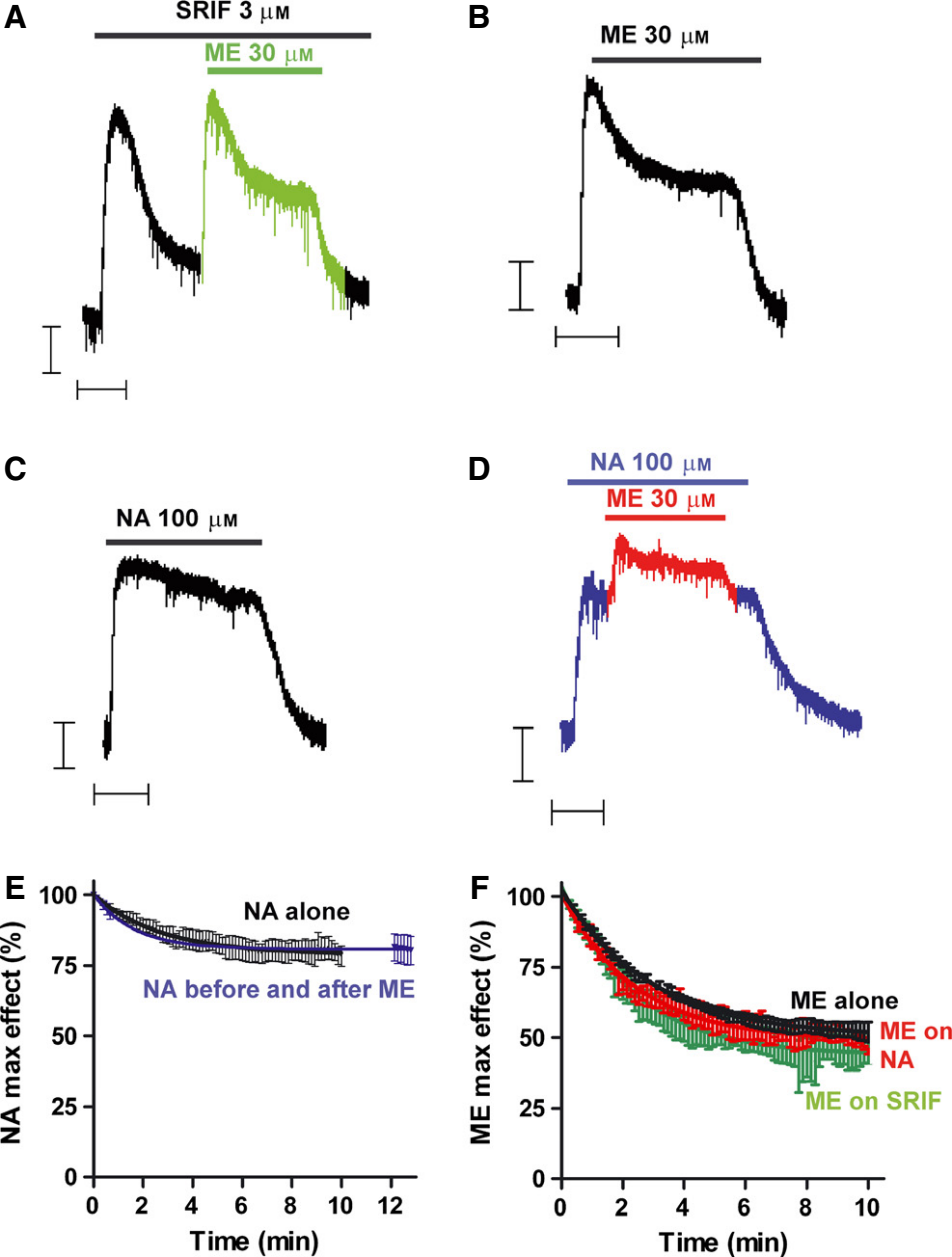
Desensitization of GPCR-evoked GIRK channel currents in mature rat LC neurons: desensitization of one GPCR type does not influence the desensitization of another. (A) Outward potassium current recorded from a single LC neuron in response to application of maximally effective concentrations of somatostatin (SRIF; 3 μm) and Met-enkephalin (ME; 30 μm). Drugs were applied for the periods indicated by the bars. The response to SST desensitized in the continued presence of the drug but application of ME in the presence of SST (shown in green) still evoked the maximal current and the response still desensitized. Scale bars represent 50 pA and 5 min. (B) Recording from another LC neuron showing desensitization of the ME response when it was applied alone. (C) A maximally effective concentration of noradrenaline (NA; 100 μm) evoked an outward current that desensitized less than that resulting from SST or ME application (compare C with A or B). (D) Application of ME in the presence of NA. The maximum NA current was slightly less than that evoked by ME (see also Bailey *et al*., 2009) so application of ME (shown in red) ‘on top’ of NA evoked a small additional current that desensitized in the continued presence of ME. On washout of ME the NA-evoked current returned to the pre-ME level. (E) The desensitization of the NA-evoked current was unaffected by the application of ME. On washout of ME the NA current (in blue) was of a similar amplitude as in cells not exposed to ME (in black) (*n* = 4 for each). (F) When considered in isolation from any underlying current the ME-evoked current desensitized with the same kinetics and to the same extent in the absence (in black) and presence of SRIF (green) or NA (red) (*n* = 4 for each).

The GIRK currents evoked by both SRIF and ME desensitized to a greater extent than the current evoked by NA (Fig. 2A–C). In the presence of SRIF, when the evoked GIRK current had desensitized, subsequent application of ME still evoked a GIRK current such that the amplitude of the combined SRIF- and ME-induced current was similar to that observed with ME alone in cells not exposed to SRIF, i.e. heterologous desensitization had not occurred (compare Fig. 2A and B). Given that MOPr and SRIF receptors couple to the same set of GIRK channels, the decay of the SRIF-evoked current cannot be due to GIRK channel inactivation as that would have reduced the response to ME. Furthermore, in the presence of SRIF, the rate and extent of the subsequent desensitization of the ME-evoked current was unchanged from that observed in cells exposed only to ME (Fig. 2F) (at 10 min, *t*_6_ = 0.91, *P* = 0.40).

If activation of one type of GPCR recruited GRK to the plasma membrane and this resulted in free βγ subunit sequestration (Raveh *et al*., 2010) then it might be expected that this would result in the heterologous desensitization of any response being evoked simultaneously through another GPCR activating the same GIRK channels. We therefore examined the effect of MOPr activation on the desensitization of the current evoked through α_2_-adrenoceptors. Application of ME, in the presence of NA, however, did not result in any further desensitization of the α_2_-adrenoceptor-mediated response (Fig. 2D and E). Furthermore, although the amplitude of the ME response ‘on top’ of the NA response was smaller than that for ME alone, as expected because the maximum currents do not summate (Fig. 1), the rate and extent of the ME-induced desensitization was unchanged (Fig. 2F) (at 10 min, *t*_6_ = 1.26, *P* = 0.26).

**FIG. 3:**
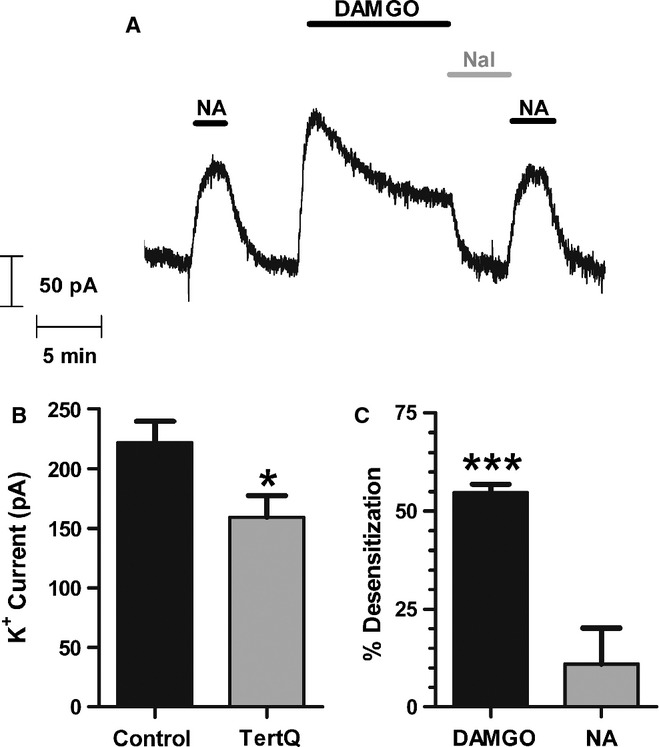
MOPr desensitization in mature LC neurons is not at the level of the GIRK channel. (A) In the continued presence of the GIRK blocker rTertiapinQ (100 nm), outward potassium current was recorded from a single LC neuron in a brain slice from a mature rat in response to application of a submaximal concentration of noradrenaline (NA; 5 μm) or a maximally effective concentration of DAMGO (10 μm), reversed with the MOPr antagonist naloxone (Nal; 5 μm). The response to DAMGO desensitized in the continued presence of the drug, whereas the response to NA which was applied both before and after the DAMGO treatment was unchanged. Pooled data from experiments as in A show that (B) the mean peak amplitudes of DAMGO-elicited potassium currents were significantly inhibited by the presence of rTertiapinQ (*P < 0.05 vs. control, Student's *t*-test; *n* = 5–7) and that (C) whereas the DAMGO response rapidly desensitized [**P < 0.001, one-sample Student's *t*-test (*t*_6_ = 26.08, *P* < 0.0001), *n* = 7], there was no significant decline in the NA response after DAMGO-induced MOPr desensitization (*P* > 0.05, *n* = 7).

**FIG. 4:**
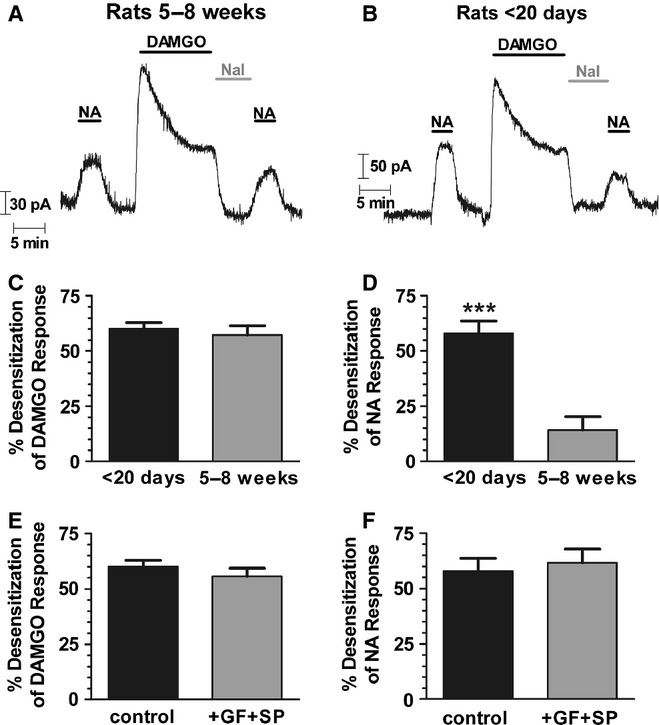
MOPr desensitization in mature LC neurons is homologous, whereas in immature LC neurons it is heterologous. (A) Outward potassium current recorded from a single LC neuron in a brain slice from a rat (> 5 weeks old) in response to application of a submaximal concentration of noradrenaline (NA; 5 μm) or a maximally effective concentration of the MOPr agonist DAMGO (10 μm), reversed with the MOPr antagonist naloxone (Nal; 5 μm). The response to DAMGO desensitized in the continued presence of the drug, whereas the response to NA which was applied both before and after the DAMGO treatment was unchanged. (B) Potassium currents recorded from an LC neuron from an immature rat (< P20) also showed rapid desensitization to DAMGO (10 μm), whereas the response to NA (5 μm) was decreased after DAMGO treatment. (C) Pooled data from experiments as illustrated in A and B showing that in LC neurons from both older rats (*n* = 5) and young rats (*n* = 6) the DAMGO response exhibited similar levels of desensitization. (D) Pooled data from experiments as illustrated in A and B showing that in LC neurons from older rats (*n* = 5) there was no significant desensitization of the NA response (*P* > 0.05) following exposure to DAMGO, whereas in neurons from young rats (*n* = 6) significant heterologous desensitization of the response to NA after DAMGO treatment was observed (***P < 0.001, Student's *t*-test). (E) Pooled data from LC neurons from < P20 rats as illustrated in B. Rapid desensitization to DAMGO (10 μm for 10 min) was unchanged by the combined presence of the PKC inhibitor GF109203x (1 μm) and the JNK inhibitor SP600125 (30 μm) (*n* = 6). (F) Pooled data from LC neurons from < P20 rats as illustrated in B. In < P20 rats, the response to NA (5 μM) was decreased after DAMGO treatment, an effect that was unchanged by the combined presence of GF109203x (GF) and SP600125 (SP) (*n* = 5–6).

Further evidence that this form of MOPr desensitization is homologous and at the level of the receptor was obtained in experiments using the selective GIRK channel inhibitor rTertiapinQ (Jin & Lu, 1999). Slices were incubated in sufficient rTertiapinQ (100 nm) to decrease the maximum response to the stable, high-efficacy MOPr peptide agonist DAMGO (sample trace Fig. 3A, pooled data Fig. 3B, *t*_10_ = 2.38, *P* = 0.04). Thus, any GIRK channel reserve is removed, and the level of GIRK channels is now the rate-limiting step in the MOPr response. If desensitization of MOPr-evoked GIRK currents was due to GIRK inhibition, the presence of rTertiapinQ would be expected both to increase the level of homologous MOPr desensitization and to reveal heterologous desensitization. However, the decline in DAMGO response over 10 min was identical in untreated (54.7 ± 2.1%, *n* = 5) and rTertiapinQ-treated (57.3 ± 4.2%, *n* = 7) slices (compare Fig. 3C with Fig. 4C; *t*_10_ = 0.49, *P* = 0.64), and after rTertiapinQ the desensitization was still homologous, as the response to a submaximal concentration of NA (5 μm) was unchanged after DAMGO-induced MOPr desensitization (Fig. 3A and C; *t*_6_ = 1.20, *P* = 0.28).

We next sought to explain why we and others observe that MOPr desensitization in LC neurons is largely homologous (Christie *et al*., 1987; Harris & Williams, 1991; Osborne & Williams, 1995; Alvarez *et al*., 2002; Bailey *et al*., 2004) whereas Blanchet & Lüscher (2002) reported that it was heterologous. While each group used fairly similar experimental approaches, Blanchet and Lüscher used brain slices from young rats (P10–21) whereas others have used slices from more mature animals (> 5 weeks old), raising the possibility that the mechanism of desensitization changes with neuronal development. We therefore used a similar experimental protocol to that of Blanchet & Lüscher (2002) and compared the nature of the MOPr desensitization induced by DAMGO in slices prepared from young and relatively mature rats (Fig. 4).

The amplitude of the GIRK current evoked by a maximally effective concentration of NA (100 μm) was the same in slices prepared from young (< P20) and older (> 5 weeks) animals (data not shown). In contrast, the response to a submaximal concentration of NA (5 μm) was greater in slices from young than older animals (compare Fig. 4A with B), suggesting a greater level of α_2_-adrenoceptor expression or receptor–effector coupling in LC neurons contained in slices from young animals. In slices from older animals the response to DAMGO (10 μm), after a 10-min application, decreased by nearly 60% (57.3 ± 4.2%, *t*_4_ = 13.71, *P* = 0.0002) from its initial peak value (Fig. 4). However, the DAMGO application did not decrease the response to a submaximal concentration of NA (5 μm) (Fig. 4A and D). The slight inhibition of the NA response in Fig. 4D (14.1 ± 6.2%, *t*_4_ = 2.27, *P* = 0.09) did not reach statistical significance (*P* > 0.05; Student's *t*-test). In contrast, in slices taken from < P20 rats application of DAMGO (10 μm) for 10 min decreased the subsequent response to both DAMGO and NA by nearly 60% [DAMGO, 60.0 ± 2.9% (*t*_5_ = 20.74, *P* < 0.0001); NA, 57.9 ± 5.7% (*t*_5_ = 10.11, *P* = 0.0002)] (Fig. 4B–D). Thus, DAMGO-induced MOPr desensitization is heterologous in slices prepared from young animals but is homologous in slices prepared from more mature animals.

Both protein kinase C (PKC) (Bailey *et al*., 2004, 2006, 2009) and c-Jun N-terminal kinase (JNK) (Melief *et al*., 2010) have been proposed as mediators of homologous MOPr desensitization. To investigate whether these kinases were involved in the heterologous desensitization observed in slices prepared from young animals, the response to NA (5 μm) was measured before and after DAMGO (10 μm for 10 min), in the combined presence of the PKC inhibitor GF109203x (1 μm) and the JNK inhibitor SP600125 (30 μm). In slices taken from animals < 20 days old, the presence of GF109203x (GF) and SP600125 (SP) did not affect the DAMGO-induced decline in either the DAMGO or the NA response (Fig. 4E and F; DAMGO response: *t*_10_ = 0.94, *P* = 0.37, NA response: *t*_9_ = 0.42, *P* = 0.68).

**FIG. 5:**
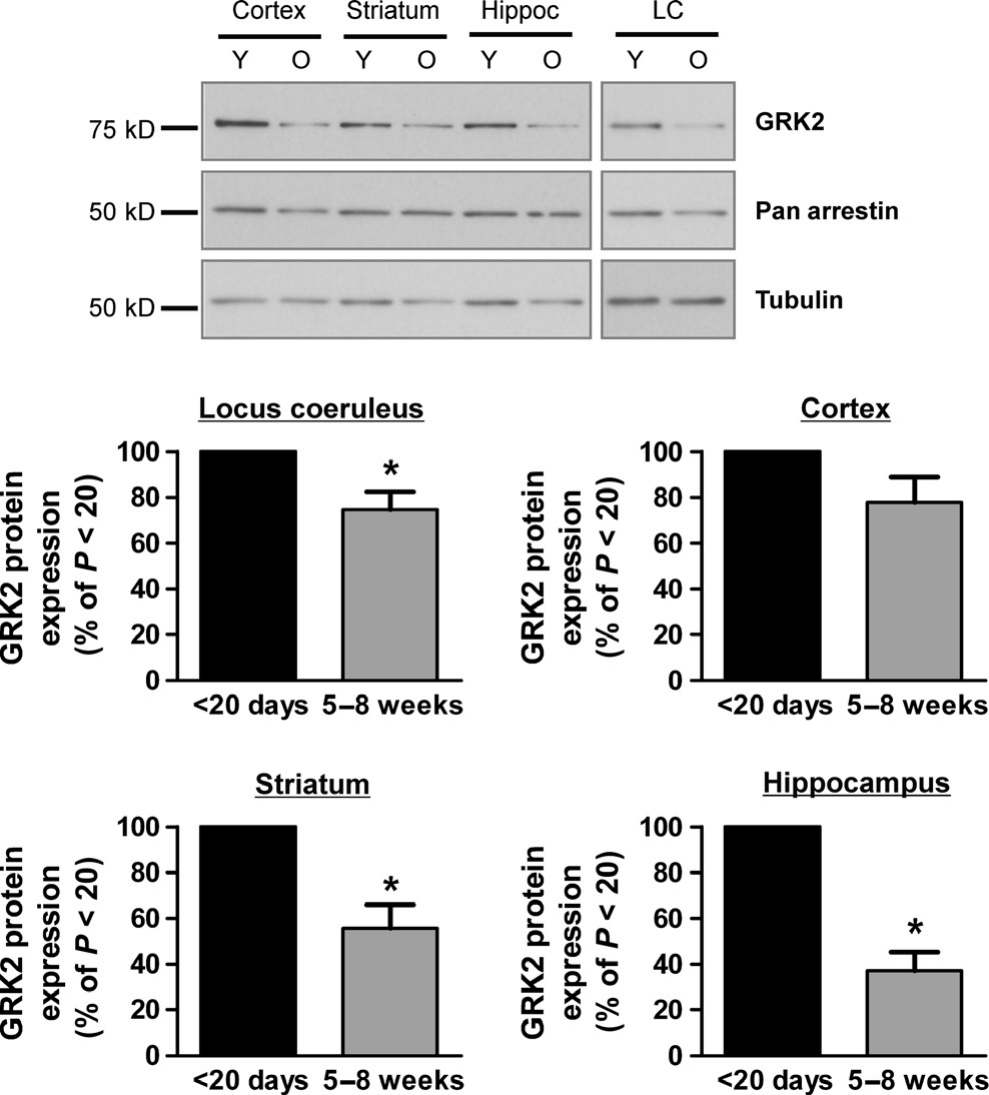
GRK2 expression levels in brain regions from young and mature rats. Protein obtained from relevant brain regions was subjected to SDS-PAGE and Western blotting with a GRK2 antibody. Illustrated for each brain region are representative blots of GRK2 from young (Y; < P20) and older (O; > 5 weeks) rat brains. Relative GRK2 levels were obtained by densitometry and normalized to the corresponding tubulin value (LC *n* = 5, cortex *n* = 9, striatum *n* = 7, hippocampus *n* = 4; GRK2 expression level in younger animals taken as 100%). *P < 0.05.

Given that Raveh *et al*. (2010) have suggested that heterologous desensitization of MOPr-activated GIRK current results from GRK sequestration of free G protein βγ subunits, we next examined whether the levels of GRK2 expression changed with age as previously reported for whole brain by Penela *et al*. (2000). In all brain regions examined the levels of GRK2 expression were higher in tissue taken from young animals (Fig. 5). The percentage GRK2 expression in mature compared with younger animals (taken as 100% in each case) was 75 ± 8% (LC) (*n* = 5), 78 ± 11% (cortex) (*n* = 9), 56 ± 10% (striatum) (*n* = 7) and 37 ± 8% (hippocampus) (*n* = 4). In the LC, GRK2 expression in striatum and hippocampus was significantly lower in older animals than younger animals (*t*_4_ = 3.23, *P* = 0.03; *t*_6_ = 4.37, *P* = 0.005; *t*_3_ = 7.83, *P* = 0.004, respectively); in the cortex the effect did not reach significance (*t*_8_ = 2.02, *P* = 0.08). Arrestin expression levels in all brain regions were unchanged between younger and older animals. Arrestin expression in mature compared with younger animals was: 100 ± 15% (cortex) (*n* = 9), 91 ± 8% (striatum) (*n* = 7) and 105 ± 9% (hippocampus) (*n* = 4) and 122 ± 46% (LC) (*n* = 5).

## Discussion

Locus coeruleus neurons provide a convenient model on which to study MOPr desensitization, in part because they are fairly homogeneous with regard to their expression of MOPrs and because they do not themselves express δ opioid receptors (DOPrs) or κ opioid receptors (KOPrs). Thus, the responses observed with opioid ligands can be assumed to be mediated only by MOPr.

There are several experimental observations on MOPr desensitization in mature LC neurons that do not fit with the βγ sequestration by the GRK model of desensitization proposed by Raveh *et al*. (2010). First, in LC neurons over-expression of a kinase-deficient dominant negative mutant (DNM) GRK that would still sequester free βγ subunits reduced rather than enhanced DAMGO-induced MOPr desensitization (Bailey *et al*., 2009). In contrast in HEK 293 cells, Raveh *et al*. (2010) observed that DNM GRK expression enhanced desensitization. However, we have previously reported that in HEK 293 cells expression of a DNM GRK reduced DAMGO-induced desensitization (Johnson *et al*., 2006). Second, in the presence of a concentration of SRIF that substantially desensitized the GIRK current in LC neurons, subsequent application of ME still evoked a GIRK current equal to that observed in cells not exposed to SRIF and the ME-evoked current in the presence of SRIF desensitized in a manner identical to that observed in cells exposed only to ME. Third, in LC neurons application of ME in the presence of NA (to activate α_2_-adrenoceptors) did not result in desensitization of the α_2_-adrenoceptor-mediated response as might be expected if MOPr activation recruited GRK to the receptor-channel complex, thus sequestering free βγ subunits released by activation of α_2_-adrenoceptors.

In addition, our findings from LC neurons taken from mature animals do not fit with the model that GIRK channel block is the mechanism underlying either homologous MOPr desensitization or MOPr-induced heterologous desensitization (see Blanchet & Lüscher, 2002), as, under conditions where the GIRK channel reserve was removed, homologous MOPr desensitization was not enhanced, nor was heterologous desensitization revealed.

Although in LC neurons from mature animals we consistently failed to see significant heterologous desensitization, profound heterologous desensitization was seen when recording from LC neurons from young (< P20) animals. A potential explanation for why desensitization changes from being heterologous to homologous as animals mature would be that the relative levels of expression of the proteins involved in desensitization change with development. If heterologous desensitization did occur due to sequestration of free βγ G protein subunits by GRK, then any increase in the GRK/βγ ratio would increase the likelihood of heterologous desensitization being observed. Penela *et al*. (2000) have previously reported that in the rat brain GRK2 levels increase up to a maximum at P20 and then decline as the animal reaches adulthood, whereas levels of arrestin 2 increase from birth to adulthood. We have observed that the levels of GRK2 were significantly higher in several brain regions including the LC of young rats. These observations are compatible with the hypothesis that in young animals MOPr desensitization is heterologous because higher levels of GRK expression allow GRK to act non-enzymatically to sequester free βγ subunits and subsequently reduce GIRK channel activation. In contrast, in mature animals MOPr desensitization is homologous because the levels of GRK are lower, presumably to levels where βγ subunit sequestration does not occur, and GRK acts through its conventional phosphorylation-dependent mechanism. To further support this hypothesis, heterologous desensitization of MOPrs has also been observed in cultured neonatal dorsal root ganglion neurons (Tan *et al*., 2003; Walwyn *et al*., 2006; Tan *et al*., 2009). Recently, Dang *et al*. (2012) have reported that in mouse LC neurons desensitization to methionine enkephalin is initially homologous but becomes heterologous with prolonged MOPr activation. They suggested that the heterologous desensitization component involved β-arrestin 2, ERK1/2 and c-Src.

We, and others, have previously shown that MOPr desensitization can occur by mechanisms other than GRK, notably via PKC (Bailey *et al*., 2004, 2006, 2009) and/or JNK (Melief *et al*., 2010). However, we have excluded the possibility that PKC or JNK are involved in the heterologous desensitization observed in LC slices from young animals as PKC and JNK inhibitors did not prevent heterologous desensitization. A further possible explanation to account for the difference between young and old animals is that there is strong electrotonic coupling between neurons in slices taken from young animals that is largely absent from older animals (Christie *et al*., 1989). Although we did observe electrotonic coupling in younger animals, as seen by oscillations of the holding current, it is unlikely that this is the mechanism by which heterologous desensitization occurs. In our experiments, naloxone was always added at the end of DAMGO treatments, so there would be no residual MOPr-mediated GIRK currents either in the neuron directly recorded from, or in neurons electrotonically coupled to it. As such, the NA response measured at the start of the experiment should be the same as the NA response at the end of the experiment, unless some form of heterologous desensitization had occurred.

While the maximum current evoked by NA in LC neurons was similar in slices from young and old animals, the potency of NA was higher in neurons from young than old animals. This excludes the possibility that heterologous desensitization was apparent in neurons from young animals because they had a lower level of α_2_-adrenoceptor expression and thus a lower receptor reserve that might reveal an underlying heterologous desensitization. A lower receptor reserve would have been reflected in a lower potency for NA. Similarly, our experiments with rTertiapinQ demonstrate that changes in GIRK channel reserve do not reveal heterologous desensitization. Therefore, changes in MOPr reserve or GIRK reserve are unlikely to be the reason why heterologous desensitization is seen in young animals but not in old animals.

In conclusion, rapid agonist-induced MOPr desensitization in LC neurons is expressed at the level of receptor–effector coupling and can be seen either as homologous or heterologous depending upon the age of the animal. It appears likely that in cell types where expression levels of GRK are high, MOPr desensitization will appear as heterologous and the actions of GRK non-enzymatic, whereas in cell types where expression levels of GRK are low, MOPr desensitization will appear as homologous and the actions of GRK enzymatic. It remains to be seen if this phenomenon applies to other GPCRs that undergo agonist-induced GRK-dependent desensitization. Our results have impact on extrapolating findings from one cell type to another and for the same cell type at different stages of development.

## Abbreviations

aCSF: artificial cerebrospinal fluid
DAMGO: d-Ala^2^, *N*-MePhe^4^, Gly-ol-enkephalin
DNM: dominant negative mutant
DOPrs: δ-opioid receptors
GIRK: G-protein inwardly rectifying
GPCR: G protein-coupled receptor
GRKs: G-protein-coupled receptor kinases
JNK: c-Jun N-terminal kinase
KOPrs: κ-opioid receptors
LC: locus coeruleus
ME: methionine enkephalin
MOPr: μ-opioid receptor
NA: noradrenaline
PKC: protein kinase C
SRIF: somatotropin release-inhibiting factor
